# Sheep scrapie and deer rabies in England prior to 1800

**DOI:** 10.1080/19336896.2023.2166749

**Published:** 2023-01-18

**Authors:** Anthony Ness, Judd Aiken, Debbie McKenzie

**Affiliations:** a Department of Biological Sciences, University of Alberta, Edmonton, Alberta, Canada; b Centre for Prions and Protein Folding Diseases, Edmonton, Alberta, Canada; c Department of Agriculture, Food and Nutritional Sciences, University of Alberta, Edmonton, Alberta, Canada

**Keywords:** deer, prion, rabies, scrapie, sheep

## Abstract

Eighteenth-century England witnessed the emergence of two neurological diseases in animals. Scrapie, a transmissible spongiform encephalopathy, is a fatal neurodegenerative disease of sheep and goats that appears in classical and atypical forms. Reports of classical scrapie in continental Europe with described symptoms date back to 1750 in what is now western Poland. However, two major outbreaks of scrapie appeared in England prior to the 1800s. References to a sheep disease with a resemblance to scrapie first appear in Southwestern England between 1693 and 1722 and in the East Midlands between 1693 and 1706. Concurrent with the descriptions of scrapie in sheep was a neurological disease of deer first appearing in the East of England. Two 18th-century writers remarked on the symptomatic similarities between the sheep and deer neurological diseases. Multiple outbreaks of the unknown deer disease existing as early as 1772 are examined and are identified as rabies.

## Scrapie prior to 1800

Scrapie in sheep and goats is the first described transmissible spongiform encephalopathy (TSE). TSEs are fatal neurodegenerative disorders caused by prions – disease-associated misfolded proteins that replicate their isoforms using a template-like mechanism [1–3]. Scrapie and chronic wasting disease (CWD) affecting cervids are contagious among susceptible animals [4–6]. Two major classifications of scrapie are recognized: classical scrapie and atypical (Nor98) scrapie. Classical scrapie presents with hind limb ataxia and/or weakness, head tremors, behavioural changes, abnormal posture and gait, weight loss, and pruritus (skin itching) which leads to sheep rubbing against objects and losing their wool [7,p.60–71, 8–11]. Atypical scrapie is usually detected prior to onset of clinical disease by prion surveillance programmes; clinical signs are typically characterized by ataxia (often in the hind limbs), behavioural changes, and weight loss, in the absence pruritus [6,12,13].

Despite millennia of domestication of sheep and goats, scrapie appeared to emerge suddenly in the 18th century. Parry attributed the sudden appearance and establishment of scrapie in England and the Electorate of Saxony to trends of extreme inbreeding which may have genetically predisposed sheep to developing the disease [7,p.8–11]. He also noted that agricultural writings in both countries flourished at this time, increasing documented reports of disease. Leopoldt of the Lordship of Sorau (then part of the Electorate of Saxony, now in western Poland) is credited with documentation of classical scrapie in continental Europe in a 1750 agricultural guide [14,15,p.348].

**Figure 1.**
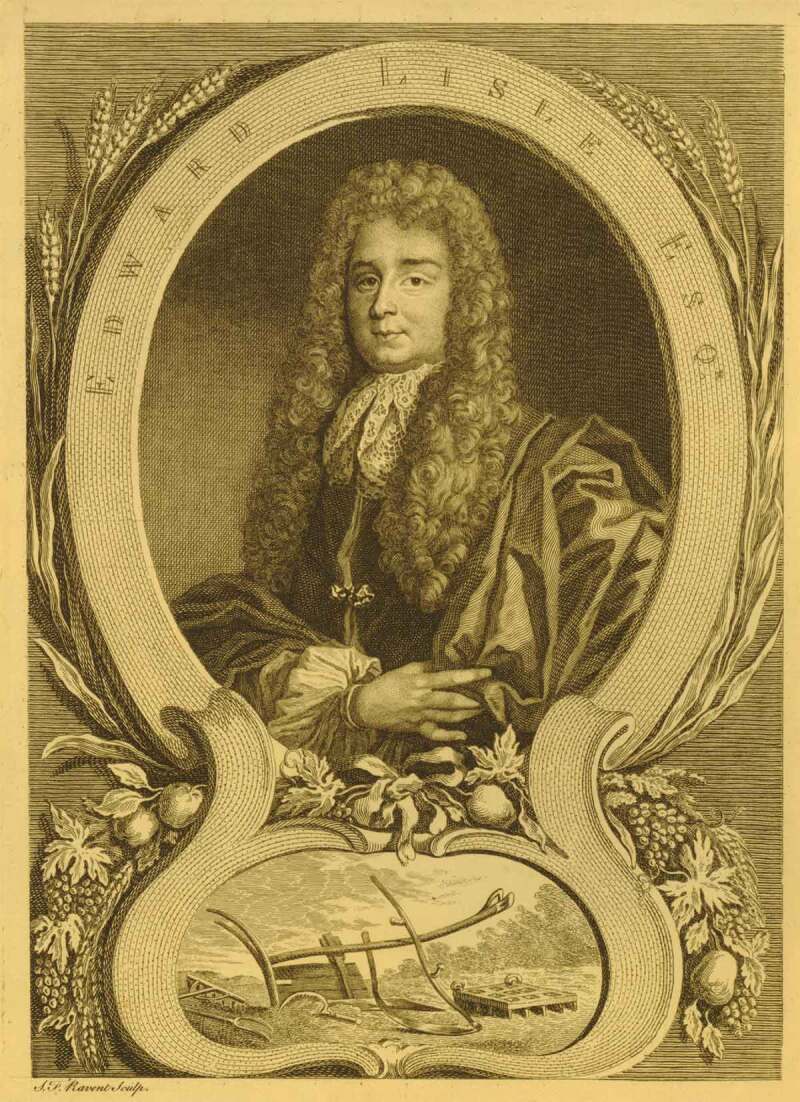
Portrait of the agriculturist Edward Lisle (b. abt. 1666–1722) on the frontispiece of ‘Observations in Husbandry’ (1757) – his posthumously compiled works. Illustrated by S. F. Ravent Sculp. Attribution: image asset no. 1546030001 from The British Museum. © The Trustees of the British Museum.

Although 1750 is often listed as the earliest dated description of sheep scrapie, the disease was likely recognized much earlier. A 1772 letter by Thomas Comber (1722–1778) [16] is frequently cited as dating scrapie in England to approximately 1732 – based on a second-hand anecdote claiming that the disease had been present for 40 years [17]. Scrapie was initially referred to a number of different names in England including *shaking*, *rickets*, *goggles* (all referring to the unsteady gait), and *rubbers* (referring to pruritic rubbing) [7,p.34–43, 14, 18,p.1–3]. An earlier description was made by Edward Lisle (Figure 1) who referred to the disease in England as the *shaking*:

Some years the sheep will be apt to be taken with a disease they call the shaking; some farms are more subject to it than others: it is a weakness which seizes their hindquarters, so that they cannot rise up when they are down: I know no cure for it.

This shaking, as I observed is incident to some farms, insomuch as some years an hundred of a flock have died of it: neither Mr. Oxenbridge, Nat. Ryalls, nor Mr. Bishop’s shepherd knew of any cure for it. – But they said that horses going with sheep are apt to cause it, and so are briery hedgerows growing out in the ground; but that milchkine [dairy cows] and goats going with the sheep were good against it … [19,p.339]

It is difficult to pinpoint when Lisle first observed *shaking* in sheep as his agricultural notes were compiled after his death, in 1722, by his son, Thomas Lisle. The time frame of Edward Lisle’s observations and notes on *shaking* is narrowed by Thomas Lisle’s foreword that states his father’s agricultural interest and study began in approximately 1693. As for location, Edward Lisle’s notes on agriculture were based on observations of farming on his various estates as well as on his travels. Lisle’s estates were in Hampshire or Wiltshire, Oxenbridge (from the above quote) farmed in Wiltshire while Ryalls and Bishop were from Dorsetshire. In a separate note regarding bloodletting for preventing a disease in sheep called red-water (babesiosis), Lisle briefly mentions that the *shaking* affects sheep in Leicestershire, the East Midlands:

[Sir Ambrose Phillipps’ shepherd] prefers bleeding in the tail to the eye-vein, both for [preventing] the red-water, and the shaking, which his sheep are subject to. [19,p.341]

The sheep affected by *shaking* in Leicestershire affected those of Lisle’s father-in-law, Sir Ambrose Phillipps of Garenton (b. Abt. 1637 – died 1706) [20,21,p.76–78], dating *shaking* in Leicestershire to 1693–1706, based on Sir Phillipps’ death. The brief mention of *shaking* in Leicestershire suggests that Lisle had first observed the disease in Wiltshire and Dorsetshire. The existence of *shaking* on multiple farms in Wiltshire and Dorsetshire suggests a more established presence of scrapie in those southern counties.

Interestingly, pruritus – causing the scratching, scraping, or rubbing of wool in classical scrapie – is not described by Lisle. By 1783, Wiltshire sheep affected by scrapie (then termed *goggles*) were described as having hind limb weakness with no mention of pruritus [22]. In the 20th and 21st centuries, pruritus is not always the dominant clinical sign and hind limb ataxia is negatively correlated with pruritus [8–10,23]. M’Gowan and Parry’s historical reviews of scrapie included non-pruritic scrapie being predominant in the South of England before 1800 [7,p.34–35, 18,p.3–4]. One explanation is that the Wilkshire and Dorsetshire cases represent a strain of scrapie resembling atypical Nor98 where pruritus is absent and hind limb ataxia can be present (although hind limb ataxia can also be present in classical scrapie) [10,13]. Nor98 is, however, poorly transmitted [6,13,24], whereas Lisle noted outbreaks. Apparent outbreaks of a Nor98-like disease could be explained by the extreme inbreeding of the Wiltshire Horn and Dorset Horn sheep conferring a high degree of genetic susceptibility to scrapie [7,p.8–11, 10, 13]. A non-pruritic form of classical scrapie is more consistent with the reported spread of *shaking* and *goggles* in the South of England. Lisle also lists the *shaking* as a disorder distinct from diseases that could be symptomatically conflated with scrapie – notably *gid* or *giddiness* (coenurosis), and the *staggers* (hypomagnesaemia) [19,p.338–339]. The symptoms described are not consistent with a differential diagnosis of rabies in sheep (a possibility at the time) which typically manifests, in sheep, as the furious form of rabies with headbutting, aggression, drooling, head and muzzle tremors, and finally paralysis with rapid death occurring within days of onset of clinical signs [25–28]. Scrapie was sufficiently predominant in Dorsetshire and Wiltshire such that, in the second half of the 18th century, it was referred to as the *Wiltshire disorder* [22, 29, 30,p.26–27]. The decline in popularity and near extinction of the Wiltshire Horn breed of sheep has been partly attributed to their reputation for developing scrapie [30,p.26–27, 31].

**Figure 2.**
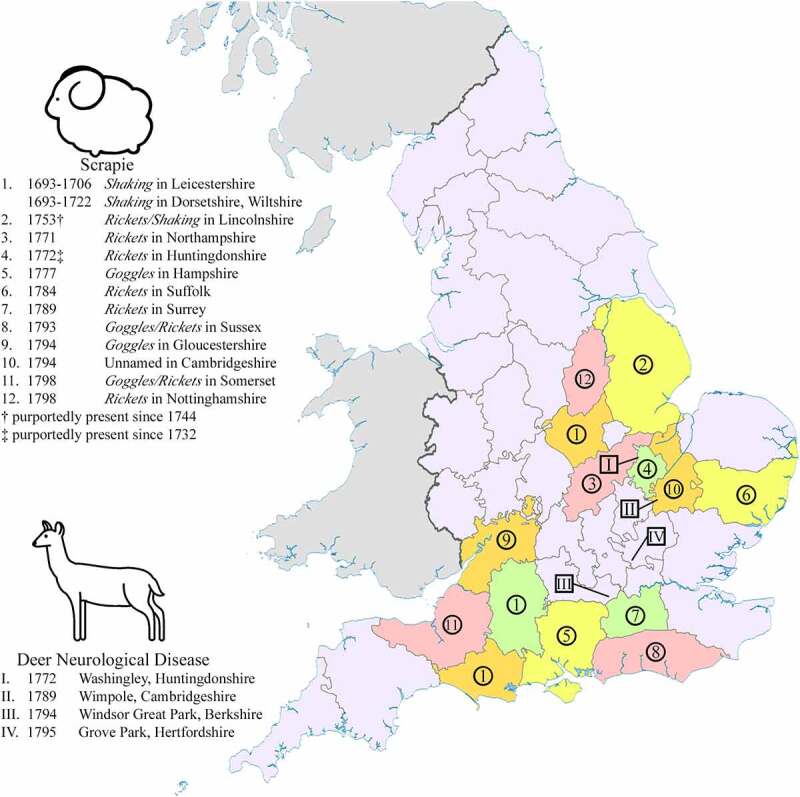
First known presence of scrapie and deer rabies outbreaks in English counties prior to 1800.

Several more cases of scrapie were reported in England prior to 1800 (Figure 2). The emergence of scrapie threatened the reputations and incomes of those involved. In 1754, Lincolnshire sheep breeders and feeders conveyed their concerns to legislators on the spread of scrapie in the area by breeders and jobbers [intermediary sellers] with the hopes of future regulations being implemented [32]. Despite a lack of described symptoms, the report includes several temporal reference points:

Mr. *Nicholas Wildman*, of *Sutton*, Grasier, said, That there has been a Distemper amongst the Sheep in *Lincolnshire*, about Ten Years, called the Rickets, or Shaking; which, he believes, is spread to other Counties; and when once a Sheep has contracted this Distemper, it never recovers:

That, in the Spring 1753, the Witness bought an Hundred Sheep of a Jobber, of different sorts; and, in Two of the Sorts, there were several Sheep which were distempered … [32]

Farmers accused of selling sheep infected with the *rickets* (Northamptonshire, 1771) and *goggles* (Hampshire, 1777) took out newspaper advertisements defending the quality of their livestock [33,34]. One Northamptonshire farmer won a defamation suit in 1785 against a shepherd who had claimed the farmer’s rams were infected with *rickets*/*rubbers* [35].

Pruritic classical scrapie in England was described by rector Thomas Comber writing from Huntingdonshire, England in 1772:

The principal Symptom of the first Stage of this Distemper, is a Kind of Light-Headedness, which makes the affected Sheep appear much wilder than usual, when his Master or Shepherd as well as a Stranger, approaches him. He bounces up suddenly from his Laire, and runs to a Distance, as though he were pursued by Dogs, &c [et cetera]. These Actions seem to indicate that his Sight is affected: and I dare say, if his Eye-Balls were examined, they would appear inflamed.

In the second Stage of the Distemper, the principal Symptom of the Sheep is his rubbing himself against Trees, Posts, &c. with such Fury as to pull off his Wool and tear away his Flesh.

The distressed Animal has now a violent Itching in his Skin, the Effect of an highly inflamed Blood: but it does not appear that there is ever any cutaneous Eruption, or salutary critical Discharge. In short, from all Circumstances the Fever appears now to be at its Height.

The third and last Stage of this dreadful Malady seems to be only the Progress of Dissolution, after an unfavourable Crisis. The poor Animal, as condemned by Nature, appears stupid, separates from the Flock, *walks irregularly*, (whence probably the Name of this Disease, *Rickets*) generally lies, and eats little. These Symptoms increase in Degree till Death, which follows a general Consumption, which appears upon Dissection of the Carcase; the Juices and even Solids having suffered a general Dissolution, insomuch that the Solids have no longer any of the good Properties of Flesh, nor the Blood of its usual Colour, &c [17].

Comber reported clinical signs consistent with classical scrapie and, based on local anecdotes, suggests that *rickets* had been in England for perhaps 40 years – i.e., 1732 which had been, before the acknowledgement of Edward Lisle’s observations, suggested to be the earliest date for scrapie. References to scrapie appear between 1784 and 1798 under names of *rickets, goggles, shaking*, and *rubbers* in the counties of Cambridgeshire, Gloucestershire, Hampshire, Leicestershire, Somerset, Suffolk, Surrey, Sussex, and the counties previously reporting the disease [30,p.26–27, 36–41,p.27, 42–45]. Norfolk in the East of England likely had scrapie prior to 1800. *Rickets* was established in multiple farms in Norfolk by 1804 with one location being affected as early as 1800 [46]. John Claridge judged in 1793 that *shaking, rickets*, and *goggles* were all the same disease and in 1809 John Lawrence, an agricultural writer, wrote that Lisle’s description of *shaking* is the same disease as *goggles* [30,p.26–27, 47].

Two distinct geographical foci of scrapie existed in England prior to 1800 (Figure 2), one spanning the South West and the South East regions of England and a second encompassing the East Midlands and the East of England regions. Parry recognized these foci as Wessex and East Anglia [7,p.34–39]. Although clinical descriptions of disease are rare, sheep in Wiltshire and Dorsetshire did not display pruritus [19,p.339, 22] while those in the east often, but not always, presented with pruritus [17,36,38,40,44]. The geographic separation of scrapie signs during 18th-century England (before prolonged and extensive trade of sheep between regions) is supportive of two independent outbreaks of scrapie – atypical scrapie or non-pruritic classical scrapie in the South of England, and classical pruritic scrapie in the East Midlands and East of England. Different breeds were affected in the two foci with disease in the East Midlands and the East of England involving the Norfolk Horn and Old Hampshire breeds while scrapie predominantly affected the Wiltshire Horn and Dorset Horn breeds in the South East and South West foci [7,p.16–24, 30,p.26–27, 37, 39–41,p.27]. The geographical separation of sheep breeds could also explain the absence of pruritus in the South of England. As speculated earlier, the extreme inbreeding of the Wiltshire Horn and Dorset Horn sheep may have influenced the predominant symptoms of scrapie in the breeds. For comparison, continental European outbreaks of classical scrapie prior to 1800 were largely restricted to descendants of imported Spanish Merino breeds [7,p.32–34]. The Old Hampshire breed is now extinct and the Norfolk Horn barely survived extinction [48,49]. The modern Wiltshire Horn and Dorset Horn are among the breeds with the highest incidence of scrapie [50]. The modern Norfolk Horn population has a high frequency of the ARR (scrapie-resistant) prion protein genotype while the Wiltshire Horn has a more mixed genotype frequency [51]. These two breeds have, however, experienced population bottleneck effects due to the near extinction of the breeds suggesting that modern prion protein genotype frequencies may not be representative of their historical populations.

## Rabies in English deer prior to 1800

Concurrent with the establishment of scrapie in England, there were reports of a disease in deer with scrapie-like symptoms. Comber, who reported the pruritic symptoms of scrapie, documented the disease in deer as follows:

I will conclude, Sir, this long Letter, by observing that there is acknowledged a strong Analogy betwixt Sheep and Deer. I am assured by several Persons of Credit, that a Distemper exactly the same as *Rickets* in Sheep is found to have arisen of late Years among Deer in some Parks (Particularly in that of – Apprice, Esq; at *Washingley*, in this County). How desirable is it, that the Masters of Parks should instruct their Keepers to observe all the Symptoms of Deer thus dying, and compare them with those of Sheep! [17]

Comber’s suggestion that there might be a link between sheep *rickets* and the deer disease leads to the question as to whether this deer disease is an unrecorded outbreak of CWD in the English countryside (Figure 2), nearly 200 years prior to the disease being described in North America [52]. The possibility of a scrapie-initiated CWD epidemic in Georgian era England is remotely possible. Sheep scrapie is transmissible to elk and white-tailed deer by intracerebral inoculation and the clinical signs of CWD are visually similar to scrapie [4,53,54]. Given the independent emergence of CWD in Colorado and Northern Europe [52,55], CWD may not be restricted to a disease of the 20th and 21st centuries. In 1794, Charles Vancouver (1756–1815?) [56] reports neurological disease in deer at nearby Wimpole (Wimple) park, Cambridgeshire:

Wimple park, contains about four hundred acres, and is at present, depastured by deer, sheep, and cow cattle; amongst the former, a disease does, and has prevailed for some years past, which in some degree, may be compared, from its resemblance with the very extraordinary one, observed amongst the sheep, in the neighbourhood of Ashley. The first symptom of the disorder, observable in the deer, is similar to that amongst the sheep; which is an apparent uneasiness in the head, and the rubbing of its horns against the trees, (this action however is common to deer, at particular seasons, in all countries, whether in a perfectly wild, or more domesticated state) but the most extraordinary effect of this disease is, that the animal appears to labour under a sort of madness, in pursuing the herd, which now flee before him, and endeavour to forsake him; trying to bite, or otherwise annoy them, with all his strength and power, which soon being exhausted, he becomes sequestered from the rest of the herd, and in that deplorable state of the disease, breaks his antlers against the trees, gnaws large collops of flesh, from off his sides, and hind quarters, appears convulsed for a short time, and soon expires.

The greater part of the flock of deer, which were very numerous in this park, have been carried off by this dreadful disorder, in the course of the last three years. In the months of July, August, and September, and when in full pasture, they are more subject to its fatal influence, than at other times, though it prevails to a certain degree throughout the year. [57]

Both Comber and Vancouver commented on the similarity of the deer disease with scrapie, suggesting they were referring to the same deer disease. The deer of the Windsor Great Park were reported as infected circa 1794, with the outbreak lasting until at least 1798 [58,p.275–276, 59]. With the disease affecting the deer in a Royal Park, King George III sought out information about the Wimpole outbreak. A 1794 letter from Philipe York, 3rd Earl of Hardwicke, the owner of the Wimpole estate, to King George III of England provides more details about the Wimpole deer disease:

It began in the summer of 1789, and principally affected the old bucks and does, the greater part of which were destroyed by it in the course of that year. Those that were attacked by the disorder, separated themselves from the herd, and ran with great violence against trees or whatever was in their way. Before the summer of 1790, upwards of 200 deer had died of the disorder out of 300 that formed the original stock … about 150 new deer were introduced into the Park in the course of that & the following year. From the year 1790, the disorder has never raged in so violent a manner; but from ten to thirty of different ages, & of the new deer as well as the old, have died ever[y] year since that time … I forgot to mention that the disorder has affected fawns of 3 or 4 days old & of the new stock: they appear to lose the use of their hind limbs, & died in a few hours … [58,p.275-276]

The described symptoms differ from CWD in the 20th and 21st centuries – notably the biting of other deer and pruritus which are both absent in clinical CWD [4]. A writer in 1799 responded to Vancouver’s report and suggested that the disease at Wimpole was caused by the *staggers* (hypomagnesaemia) despite pruritus and biting also not being a symptom of the latter [60,p.229–237, 61,62]. CWD can be transmitted vertically, but is not immediately lethal to fawns [63,64].

Further outbreaks of the unknown deer disease would continue to periodically appear in deer parks into the 19th century. The most thorough investigation into the disease outbreaks was published in 1888. Cope and Horsely published a combined report on the 1886 outbreak in Richmond Park. Cope investigated the case history while Horsely studied the disease experimentally. Cope’s report provides evidence of deer disease outbreaks with the same symptoms in Grove Park, Hertfordshire in 1795, the Windsor Great Park outbreak mentioned earlier, and cases on other estates in 1872 and 1880 [28,59]. The investigators were not aware of the older Washingley and Wimpole cases. One of the earliest symptoms noted in the Cope report is infected deer holding their noses up to the air – a symptom reminiscent to the raised head and fixed stare of scrapie-infected sheep [7,p.61, 59]. Symptoms in all of the outbreaks were nearly identical to those reported at Wimpole – excessive rubbing of vegetation (sometimes so extreme that the bone of the forehead was exposed), aggressive behaviour, chasing and biting other deer, and biting of their own sides. Like Wimpole, a fawn from Richmond Park became symptomatic and rapidly died.

Affected, penned deer displayed extreme aggression (including attempts at biting) towards handlers, hind limb ataxia progressing to paralysis, and death within 2–8 days of onset of clinical signs [59]. The short clinical phase of disease (within 8 days) excludes CWD prions as the causative agent. The disease persisted when Cope moved the deer to new pastures. Based on clinical signs and case history, Cope and a veterinary inspector, Lupton began to suspect rabies. Rabies is an encephalitic disease caused by the neurotropic viruses of the *Lyssavirus* genus which is most commonly transmitted through saliva from bites by infected animals [65,66]. Symptomatic animals typically present with one of two forms – aggressive (furious) or dumb (paralytic) rabies [65–67]. Cervid rabies in the modern world is, generally speaking, rare and self-limiting [68–73].

Although deer were known, on rare occasions, to be bitten and infected by rabid dogs, neither veterinarians nor the extant literature had knowledge of rabies outbreaks in deer herds. Deer were generally believed to be dead-end hosts of rabies. The feasibility of rabies transmission between cervids via biting was regarded as doubtful as deer have a dental pad instead of upper incisors. Print news reports of a possible herd of rabies-infected deer at Stainborough Park in 1856 initiated an investigation by a medical officer who, controversially, declared that the disease was rabies. Subsequent veterinarian inquirers and physicians remained unconvinced of the Stainborough outbreak being caused by rabies [74,75]. Cope did not refer to the 1856 outbreak in his historical background [59].

Cope and Horsley did, however, investigate rabies as a possible cause of the Richmond Park epizootic [59]. To understand disease transmission, an uninfected deer and a clinically affected deer from Richmond Park were co-housed in a single pen. The infected deer immediately attacked the other, biting about the ears and neck. The naïve animal developed clinical signs 19 days later and died shortly thereafter. Careful observation of Richmond Park deer determined that biting by infected deer did not cause lacerating open wounds, but the attacked deer were exposed to residual saliva when subsequently licking the bitten areas.

Cope sent infected deer to Horsley in London to experimentally test for rabies. An infected buck sent to London was too violent to approach for 2 days until it fell unconscious, dying following a high fever on the third day. Spinal cord tissue from the violent buck, other infected deer, and medullary tissue from a fawn that died of clinical disease were intracerebrally inoculated into rabbits which all developed and died of ‘typical’ rabies. Spinal cord tissue from the fawn was inoculated into a dog which also developed and died of rabies. The cause of the outbreaks in deer was, therefore, conclusively confirmed.

Similar to scrapie, rabies outbreaks in deer were unknown prior to the 18th century despite deer parks existing since medieval times. While extreme inbreeding of sheep may have created the genetic predisposition for certain breeds of sheep to develop scrapie, what could have allowed for rabies, an ancient disease, to suddenly become epizootic in deer? Rabies has been recorded in Britain since the High Medieval Period, but the disease did not become widely entrenched in England (almost exclusively in dogs) until the 1770s [67]. The presence of rabies in England does not by itself explain why rabies outbreaks in deer occurred during this time period. The multiple rabies outbreaks in English deer beginning in the late 18th century may have been influenced by two changes to deer parks. Beginning in the 18th century, typical English deer parks were transformed from vast royal forests into smaller estate enclosures with paddocks and pastures [76]. The density of trees decreased to provide the landed gentry with views of their land and ornamental herds [77]. The widespread establishment of rabies in dogs combined with the smaller enclosures and the reduced tree cover of 18th- and 19th-century deer parks may have fostered the conditions for rabies outbreaks in deer parks. The cessation of rabies outbreaks in deer can then be attributed to policies in the 1880s and 1890s aimed aggressively at eradicating rabies in dogs, with the elimination of rabies in England by 1902 [28,67].

## Conclusion

Two neurological diseases of animals emerged in 18th-century England. The first recorded appearance of the scrapie prion disease in sheep can be dated to between 1693 and 1722 in the Southwest of England and between 1693 and 1706 in the East Midlands. Thomas Comber’s letter on scrapie was published in 1772 with the concluding intrigue of an existing, scrapie-like disease in deer. The reports of diseased deer in 18th-century England by Comber and Vancouver (cases unknown to Cope and Horsley) can now be attributed not to a prion, but to rabies.
